# Monkeypox Virus Infection: A Global Warning for the Possible Next Pandemic

**DOI:** 10.34172/jrhs.2022.80

**Published:** 2022-06-28

**Authors:** Jalal Poorolajal

**Affiliations:** ^1^Research Center for Health Sciences, School of Public Health, Hamadan University of Medical Sciences, Hamadan, Iran; ^2^Department of Epidemiology, School of Public Health, Hamadan University of Medical Sciences, Hamadan, Iran

 Monkeypox is a rare zoonotic infectious disease. Monkeypox virus belongs to the *Orthopoxvirus* genus of the *Poxviridae* family which includes both the *variola* (smallpox) virus and the *Vaccinia* virus (used in the smallpox vaccine).^1^ Monkeypox was initially discovered in 1958 and occurs mostly in Central and West Africa. Monkeypox is a global public health concern because more than 120 confirmed or suspected cases of the disease were recently identified in at least 11 non-endemic countries such as North America and Europe in May 2022.^2,3^

 Several reasons have been suggested for the recent increase and spread of pandemics in the world. Based on current evidence environmental exploitation caused by land-use changes, deforestation, agriculture expansion, wildlife trade and consumption, and urbanization can disrupt natural interactions between wildlife and their microbes, as well as increase contact between wildlife, livestock, and humans, potentially resulting in new infectious diseases and pandemics around the world.^4,5^

 Monkeypox is less contagious than smallpox. The virus enters the body through broken skin, the respiratory tract, or the mucous membranes (eyes, nose, or mouth).^1^ Human-to-human transmission occurs through direct contact with lesions, body fluids, and respiratory droplets. Prolonged face-to-face contact is required because respiratory droplets can only reach a few feet. Although almost all recently diagnosed cases include men aged 20 to 50, many of whom are men who have sex with men, it is unclear at this time if monkeypox can be transmitted specifically through sexual transmission routes. Indirect contact with contaminated materials such as bedding has also been documented. The animal-to-human transmission may occur through a bite or scratch as well as direct or indirect contact with body fluids or lesion material.^2,6,7^

 Clinical presentations of monkeypox are similar to but less severe than smallpox which was eradicated in 1980. The incubation period varies from 6 to 13 days but can take from 5 to 21 days. The disease is characterized by fever, headache, myalgia, backache, chills, exhaustion, and lymphadenopathy that may initially appear similar to chickenpox, measles, and smallpox. The illness typically lasts for 2 to 4 weeks.^1,7^

 What should we do now to prevent the spread of monkeypox and the possibility of an epidemic? Based on the limited information available at present, the risk of monkeypox to the public is very low. Although containment strategies are not necessary at present time, however, the following measures that can be taken to prevent the silent spread of the disease: (a) raising public awareness of risk factors and educating people about the measures they can take to reduce their exposure to the virus; (b) anyone who has clinical manifestations of monkeypox should contact a health care provider right away; (c) isolation of suspected or confirmed cases of monkeypox; (d) hand washing with soap and water or using an alcohol-based hand sanitizer immediately after unprotected contact with wild or sick animals or suspected patients or contaminated materials; (e) avoid contact with animals that could harbor the virus or direct contact with any contaminated materials such as bedding or laundry; (f) availability of laboratory equipment for confirmatory monkeypox virus-specific testing on lesion specimens that clinicians obtain from suspected patients; (g) feasibility of access to suitable vaccine especially for high-risk people such as laboratory workers and health professionals as well as general population if necessary.^1,2,7^

 According to the current knowledge, vaccines used during the smallpox eradication program also provide protection against monkeypox. According to statistics from Africa, the smallpox vaccination is at least 85% effective at preventing monkeypox.^8^ However, routine smallpox vaccination with vaccinia-based vaccines has been stopped by all countries at least 40 years ago. Therefore, unvaccinated populations, aged under 40, are now more susceptible to monkeypox virus infection.

## Conflict of interest

 None.
